# Opioid and GABA_B_ receptors differentially couple to an adenylyl cyclase/protein kinase A downstream effector after chronic morphine treatment

**DOI:** 10.3389/fphar.2014.00148

**Published:** 2014-06-24

**Authors:** Elena E. Bagley

**Affiliations:** Discipline of Pharmacology, Sydney Medical School, University of SydneySydney, NSW, Australia

**Keywords:** opioid, GAT-1, GABA_B_ receptor, periaqueductal gray, withdrawal

## Abstract

Opioids are intensely addictive, and cessation of their chronic use is associated with a highly aversive withdrawal syndrome. A cellular hallmark of withdrawal is an opioid sensitive protein kinase A-dependent increase in GABA transporter-1 (GAT-1) currents in periaqueductal gray (PAG) neurons. Elevated GAT-1 activity directly increases GABAergic neuronal excitability and synaptic GABA release, which will enhance GABAergic inhibition of PAG output neurons. This reduced activity of PAG output neurons to several brain regions, including the hypothalamus and medulla, contributes to many of the PAG-mediated signs of opioid withdrawal. The GABA_B_ receptor agonist baclofen reduces some of the PAG mediated signs of opioid withdrawal. Like the opioid receptors the GABA_B_ receptor is a G_i_/G_o_ coupled G-protein coupled receptor. This suggests it could be modulating GAT-1 activity in PAG neurons through its inhibition of the adenylyl cyclase/protein kinase A pathway. Opioid modulation of the GAT-1 activity can be detected by changes in the reversal potential of opioid membrane currents. We found that when opioids are reducing the GAT-1 cation conductance and increasing the GIRK conductance the opioid agonist reversal potential is much more negative than *E*_*k*_. Using this approach for GABA_B_ receptors we show that the GABA_B_ receptor agonist, baclofen, does not couple to inhibition of GAT-1 currents during opioid withdrawal. It is possible this differential signaling of the two G_i_/G_o_ coupled G-protein coupled receptors is due to the strong compartmentalization of the GABA_B_ receptor that does not favor signaling to the adenylyl cyclase/protein kinase A/GAT-1 pathway. This highlights the importance of studying the effects of G-protein coupled receptors in native tissue with endogenous G-protein coupled receptors and the full complement of relevant proteins and signaling molecules. This study suggests that baclofen reduces opioid withdrawal symptoms through a non-GAT-1 effector.

## Introduction

Opioids are intensely addictive, and cessation of their chronic use is associated with a withdrawal syndrome consisting of severe early physical symptoms and late features such as craving. Relapse into drug-taking behaviors often occurs as a result of this withdrawal syndrome (Mattick and Hall, 1996; Williams et al., 2001), which is thought to result from neuronal adaptations that develop to restore homeostasis during chronic opioid exposure (Himmelsbach, 1943). On cessation of opioid administration, persistent counteradaptations in critical brain regions are unmasked and cause the withdrawal syndrome. A rebound increase of adenylyl cyclase/protein kinase A (PKA) signaling is one counteradaptation. While opioid agonists acutely inhibit adenylyl cyclase activity in the brain (Collier and Roy, 1974) and specifically in the periaqueductal gray (PAG) (Fedynyshyn and Lee, 1989), there is a compensatory increase in adenylyl cyclase signaling during chronic treatment with morphine *in vitro* (Sharma et al., 1975; Avidor-Reiss et al., 1997) and *in vivo* (Terwilliger et al., 1991) resulting in rebound hyperactivity of this cascade during withdrawal. Microinjections of PKA inhibitors into the PAG attenuate a spectrum of opioid withdrawal behaviors similar to those induced by microinjections of opioid antagonists (Maldonado et al., 1995; Punch et al., 1997). Whilst the importance of upregulated adenylyl cyclase/PKA during opioid withdrawal has been appreciated for many years we have only recently found the cellular target of PKA that results in withdrawal symptoms (Bagley et al., 2005b, 2011). We found that elevated PKA activity during withdrawal increases GABA transporter 1 (GAT-1) currents in PAG neurons (Bagley et al., 2005b). Elevated GAT-1 activity directly depolarizes and thus hyperexcites GABAergic PAG neurons and nerve terminals, which presumably enhances GABAergic inhibition of PAG output neurons (Bagley et al., 2005b). This reduced activity of PAG output neurons to several brain regions, including the hypothalamus and medulla, results in opioid withdrawal signs (Bagley et al., 2011).

Opioid receptors are G_i_/G_o_ coupled G-protein coupled receptors that inhibit adenylyl cyclase through their Gα subunit. The GABA_B_ receptor is another G_i_/G_o_ coupled G-protein coupled receptor (Bettler et al., 2004) that inhibits adenylyl cyclase activity (Gerber and Gähwiler, 1994; Kuner et al., 1999; Bettler et al., 2004; Vanhoose et al., 2004; Connelly et al., 2013). GABA_B_ receptors are expressed in almost all PAG neurons (Chieng and Christie, 1996; Margeta-Mitrovic et al., 1999; Bagley et al., 2005a). The GABA_B_ receptor agonist baclofen reduces some PAG mediated signs of opioid withdrawal in humans (Ahmadi-Abhari et al., 2001; Tyacke et al., 2010) and animal models (Bexis et al., 2001; Tyacke et al., 2010) and is used in drug cocktails for treatment of opioid withdrawal (Collis, 2008). Given the similarities in coupling and the therapeutic utility of GABA_B_ receptor agonists, in this study we ask whether GABA_B_ receptor agonists act like opioids to reduce GAT-1 activity during opioid withdrawal.

## Materials and methods

### Chronic treatment with morphine

Morphine dependence was induced by a series of subcutaneous injections of sustained-release morphine suspension into male C57B16/J mice (300 mg/kg morphine base) as in previous experiments (Bagley et al., 2005b, 2011). Injections (0.1–0.2 ml) were made under light halothane anesthesia on days 1, 3, and 5, and mice were used for experiments on days 6 or 7. Morphine base was suspended in 0.1 ml mannide mono-oleate (Arlacel A, Sigma), 0.4 ml light liquid paraffin and 0.5 ml 0.9% w/v NaCl in water. Vehicle-treated mice were injected on the same schedule with morphine-free suspension.

### Tissue preparation and recordings

PAG slices (220–250 μm) were cut from 4- to 6-week-old mice and were maintained at 34°C in a submerged chamber containing physiological saline (ACSF) equilibrated with 95% O_2_ and 5% CO_2_ and were later transferred to a chamber superfused at 2 ml/min with ACSF (34°C) for recording. The standard ACSF contained 126 mM NaCl, 2.5 mM KCl, 1.4 mM NaH_2_PO_4_, 1.2 mM MgCl_2_, 2.4 mM CaCl_2_, 11 mM glucose, and 25 mM NaHCO_3_. Brain slices from both morphine-dependent and vehicle-treated mice were maintained *in vitro* in ACSF containing 5 μM morphine. Unless otherwise stated, slices were spontaneously withdrawn by incubation in morphine-free ACSF for at least 1 h before an experiment. CGP55845 was a gift from Ciba Ltd (Basel, Switzerland).

PAG neurons were visualized using infra-red Nomarski optics. Perforated patch recordings were made using patch electrodes (4–5 mΩ) filled with 120 mM K acetate, 40 mM HEPES, 10 mM EGTA, 5 mM MgCl_2_, with 0.25 mg/ml Pluronic F-127, 0.12 mg/ml amphotericin B (pH 7.2, 290 mosmol/l). Liquid junction potentials for K acetate internal solution of −8 mV with ACSF were corrected. Series resistance (<25 MΩ) was compensated by 80% and continuously monitored. During perforated patch recordings, currents were recorded using a Axopatch 200A amplifier (Axon Instruments), digitized, filtered (at 2 kHz), and then acquired (sampling at 10 kHz) in pClamp (Axon Instruments) or using Axograph Acquisition software (Axon Instruments).

All pooled data are expressed as mean ± s.e.m. We tested for significance using the unpaired Student's *t*-test.

## Results

**Figure 1:**
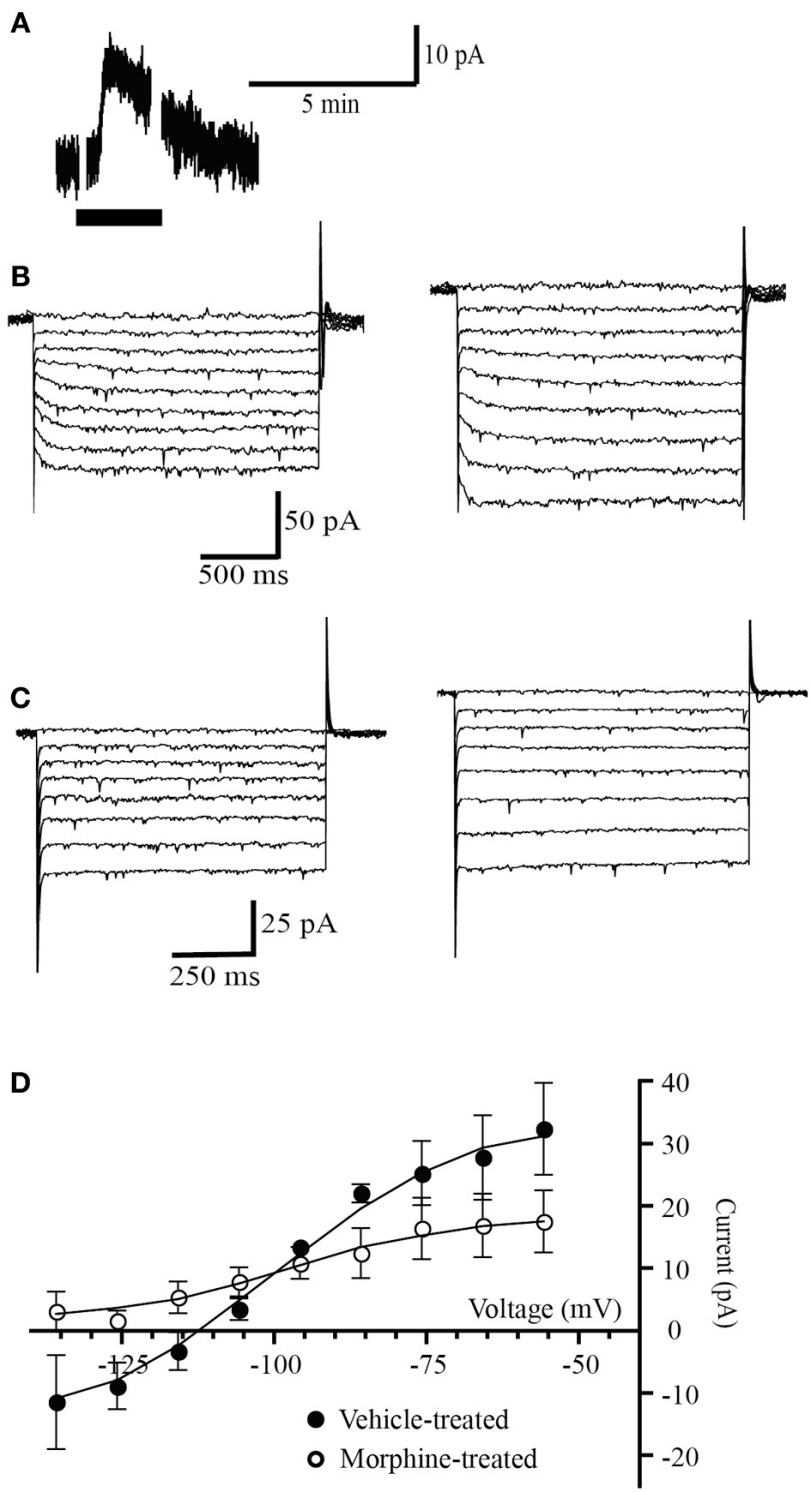
**Opioid receptors simultaneously couple to a potassium conductance and a GAT-1 conductance. (A)** Example trace of currents from a neuron voltage clamped at −56 mV (drug superfusion shown by bars). MENK (30 μM) induced an outward current in neuron from a morphine-treated mouse. **(B,C)** Currents produced by voltage steps from −56 mV to −136 mV in −10 mV increments in a neuron from **(B)** a vehicle-treated mouse and **(C)** a morphine-treated mouse before (left) and during MENK (30 μM) application (right). **(D)** Subtracted current-voltage relationships for MENK (current in MENK—current during control conditions). Reversal potentials were determined at the point where they cross the abscissa. The MENK current reversed polarity near *E*_K_ in neurons from vehicle-treated (*n* = 4), but not in neurons from morphine-treated mice (*n* = 5).

**Figure 2:**
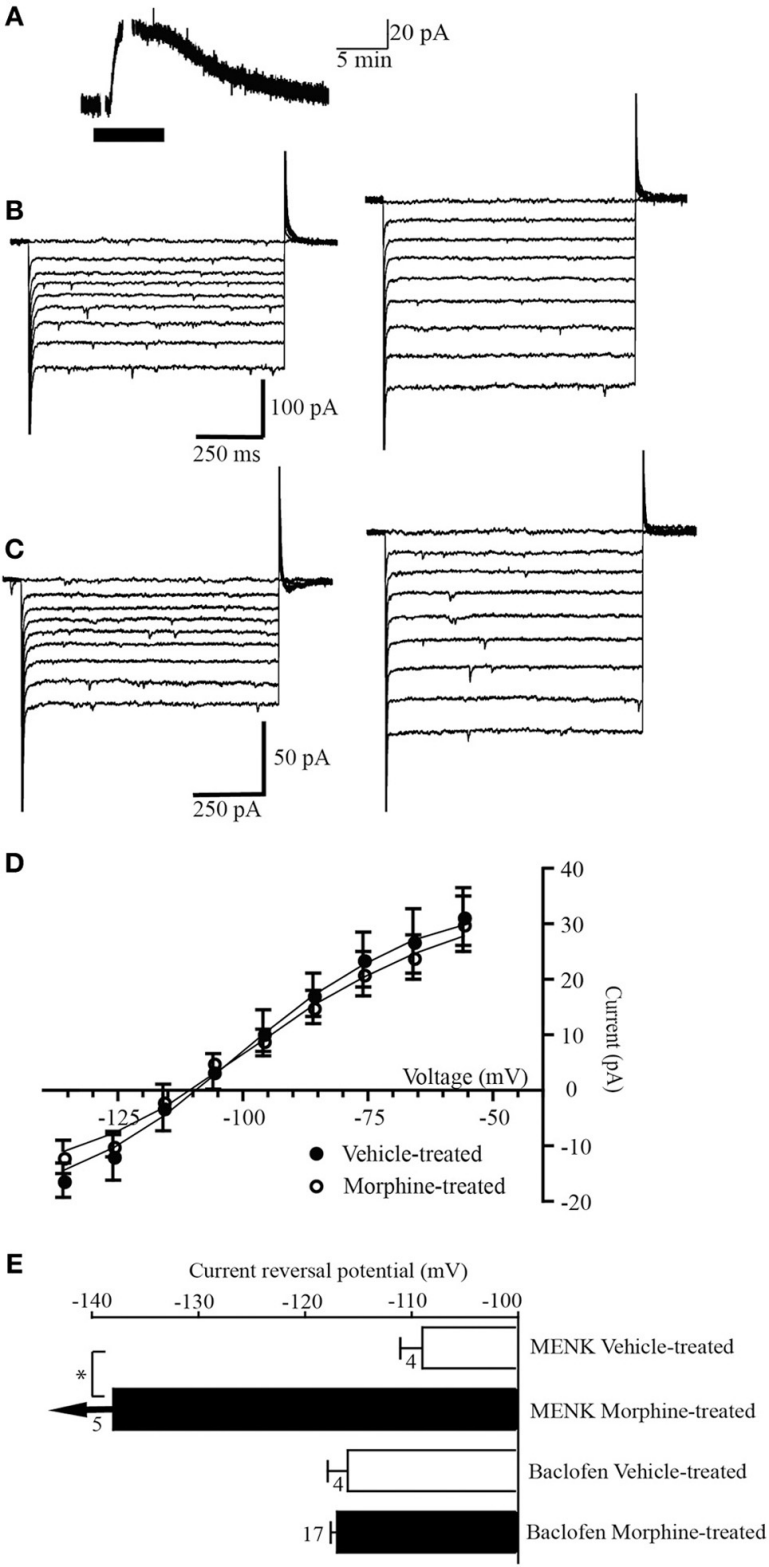
**GABA_B_ receptors do not couple to a GAT-1 conductance. (A)** Example trace of currents from a neuron voltage clamped at −56 mV (drug superfusion shown by bars). Baclofen (10 μM) induced an outward current in a neuron from a morphine-treated mouse. **(B,C)** Currents produced by voltage steps from −56 mV to −136 mV in −10 mV increments in a neuron from **(B)** a vehicle-treated mouse and **(C)** a morphine-treated mouse before (left) and during baclofen (10 μM) application (right). **(D)** Subtracted current-voltage relationships for baclofen (current in baclofen–current during control conditions). Reversal potentials were determined at the point where they cross the abscissa. The baclofen current reversed polarity near *E*_*K*_in neurons from vehicle-treated (*n* = 8), and morphine-treated mice (*n* = 17). **(E)** Reversal potential of the MENK and baclofen-induced currents in cells from vehicle-treated and chronic morphine-treated mice. Arrows indicate that the average current did not reverse polarity at the most negative potential that could be tested (−136 mV). The number of neurons is shown beside the bar. ^*^P-value of 0.034, Students *t*-test.

In PAG neurons, withdrawal from chronic morphine-treatment stimulates a protein kinase A-dependent increase of the GAT-1 cation conductance (Bagley et al., 2005b, 2011). The increased GAT-1 activity is sensitive to opioid inhibition and therefore during opioid withdrawal it can be detected by changes in the opioid agonist met-enkephalin (MENK) current reversal potential. When MENK is reducing the GAT-1 cation conductance and increasing the GIRK conductance the MENK reversal potential will be much more negative than *E*_*k*_ (Bagley et al., 2005b). Superfusion of (MENK) produced an outward current in 4 out of 6 PAG neurons voltage clamped at −56 mV in slices from vehicle-treated mice (30 ± 4 pA, *n* = 4, Figure 1A). In neurons from vehicle-treated mice the ME current reversed polarity at a potential of −109 ± 4 mV (*n* = 4, Figures 1B,D), close to the potassium reversal potential (*E*_k_, −103 mV) in these conditions as we have previously reported in mice (Bagley et al., 2005b, 2011). In neurons from chronic morphine-treated mice, the MENK-induced current reversed in only 2 of 5 cells (Figure 1C). In the neurons where the MENK current did not reverse polarity at the most negative potential tested, the reversal potentials was assigned a value of −136 mV, a conservative approach we adopted in previous studies to deal with technical inability to determine extremely negative reversal potentials (Bagley et al., 2005b, 2011). The nominal reversal potential for the 5 cells was −1.8 ± 4 mV (Figures 1C,D) that is significantly more negative than neurons from vehicle mice (*p* = 0.034, Students *t*-test, Figure 2E). We have previously shown that in the presence of the GAT-1 inhibitor, NO-711, results in ME currents that reversed polarity close to the value for MENK currents in neurons from vehicle-treated mice (Bagley et al., 2005b, 2011).

Superfusion of the GABA_B_ receptor agonist baclofen (10 μM) produced an outward current in all 8 PAG neurons voltage clamped at −56 mV in slices from vehicle-treated mice (31 ± 5 pA, *n* = 14, Figure 2A). In neurons from vehicle treated mice the baclofen current reversed polarity at a potential of −116 ± 5 mV (*n* = 8, Figures 2B,D,E), close to *E*_k_ in these conditions (−103 mV). In neurons from morphine-treated mice the baclofen-induced outward current (30 ± 5 pA, *n*= 17) reversed polarity (−117 ± 2 mV, *n* = 17, Figures 2C–E) close to *E*_k_ (−103 mV) and at a similar membrane potential to cells from vehicle-treated mice (*p* = 0.71 Students *t*-test).

## Discussion

A cellular hallmark of withdrawal in PAG is protein kinase A-dependent increases in GAT-1 currents (Bagley et al., 2005b, 2011). This study showed that even though the opioid receptors and GABA_B_ receptor are both G_i_/G_o_ coupled G-proteins coupled receptors occurring in the same neurons GABA_B_ receptors are unable to couple via PKA to the additional GAT-1 conductance. The selectivity is not due to the GABA_B_ receptor and opioid receptors being located in different neurons. Whilst only two thirds to three quarters of PAG neurons are opioid sensitive, as shown in this study and previously (Vaughan et al., 2003), almost all PAG neurons are sensitive to GABA_B_ receptor agonists (97% in this study and Chieng and Christie, 1995). In this study we only included neurons that were sensitive to both agonists. GABA_B_ receptor agonists inhibit adenylyl cyclase after expression in cell lines (Kuner et al., 1999; Bettler et al., 2004) and in brain tissue (Gerber and Gähwiler, 1994; Vanhoose et al., 2004) and there are examples where this inhibition of adenylyl cyclase regulates ionic conductances in several different brain regions (Gerber and Gähwiler, 1994; Connelly et al., 2013). Therefore, if the receptors are located on the same neurons and can couple to the adenylyl cyclase/PKA signaling pathway this suggests that there must be some sort of compartmentalization that prevents baclofen from regulating GAT-1 activity. Conversely, compartmentalization may favor opioid receptor regulation of GAT-1.

Like other G_i_/G_o_ coupled G-proteins coupled receptors GABA_B_ receptors can couple to several effectors, including calcium channels, GIRK and adenylyl cyclase. However, GABA_B_ receptors appear to show greater segregation than other G-protein coupled receptor signaling and possibly stronger regulation into nano-signaling complexes. Opioids and GABA_B_ receptors have previously been shown to differentially couple to their effectors. This occurs in GABAergic nerve terminals in the PAG. Opioid receptor activation reduces GABA release from nerve terminals in the PAG through modulation of a voltage dependent potassium channel where as GABA_B_ receptors do not couple to GABA inhibition through this mechanism (Vaughan et al., 1997). It seems likely that, as in this study, both the GABA_B_ receptors and opioid receptors are expressed on the same GABAergic terminals in the PAG again indicating compartmentalization. This also occurs outside the PAG. In locus coeruleus neurons both opioid and α2 receptors inhibit the same population of calcium channels in these neurons but GABA_B_ receptors inhibit a separate population of calcium channels in the same cell (Chieng and Bekkers, 1999). Of all the G_i_/G_o_ coupled effects there is evidence that GABA_B_ receptor regulation of AC/PKA activity may be particularly affected by compartmentalization. GABA_B_ receptor inhibition of adenylyl cyclase in the hippocampus was stimulation dependent whereas other G_i_/G_o_ coupled receptors were able to inhibit adenylyl cyclase regardless of how it was stimulated (Vanhoose et al., 2004). These differences may be due to GABA_B_ receptors being localized to nano-signaling complexes that influence their signaling to different effectors. The influence of nan-signaling complexes on GABA_B_ receptors on signaling is evidenced by inclusion of GABA_B_ receptors in nano-signaling complexes facilitating inhibition of calcium channels but not inhibition of adenylyl cyclase (Laviv et al., 2011). Further, GABA_B_ receptors, and especially the splice variants of the GABA_B1_ receptor, show differential subcellular localization and associations with protein clusters that alter their coupling to effectors (Vigot et al., 2006). Therefore, GABA_B_ receptors may not modulate GAT-1 activity in this study because they are preferentially associated with proteins or located in regions of the cell that do not favor inhibition of adenylyl cyclase. Whilst the evidence for strong compartmentalization of GABA_B_ receptor signaling is convincing it is also possible that it is the location/compartmentalization of the opioid receptors that allows their coupling to GAT-1 activity. Perhaps opioid receptors are more closely localized to the subcellular region or associated with the adenylyl cyclase/PKA/GAT-1 proteins upregulated by chronic opioid inhibition.

If we want to ask questions about processes occurring in the brain during particular disease states, as opposed to cell lines or cultured cells, it is critical that these experiments are conducted in native tissue. Experiments in native tissue study endogenous GPCRs with the full complement of relevant proteins and signaling molecules. In fact the opioid-AC-PKA modulation of GAT-1 was a surprise because the only consensus site for PKA phosphorylation is extracellular (Guastella et al., 1990) making it an unlikely candidate for regulation by PKA. Whilst the effect of PKA activation on the GAT-1 transporter has not been comprehensively studied, the subcellular location, enzymatic activity, and absolute level of GAT-1 is regulated in a complex inter-related manner by PKC activity, GABA concentration, ionic conditions, tyrosine kinase activity, and the release protein syntaxin 1A (Beckman and Quick, 1998; Beckman et al., 1998; Whitworth and Quick, 2001; Quick, 2002). Therefore, it is likely due to AC/PKA altering GAT-1 activity through an intermediary protein not expressed/or active in the cultured cells. Support for this proposal comes from another study of GAT-1 in brain tissue showing that AC/PKA activity facilitates GAT-1 transport by curbing tonic PKC-mediated inhibition of GAT-1 activity and cell surface expression (Cristóvão-Ferreira et al., 2009). Therefore, during opioid withdrawal the overshoot in AC/PKA activity in PAG neurons could be could indirectly increasing GAT-1 activity through reducing PKC restraints on GAT-1 activity. The results from this study also show how important it is to conduct experiments in native tissue. We would have predicted that another G_i_/G_o_ coupled GPCR, such as GABA_B_, that inhibits adenylyl cyclase (Gerber and Gähwiler, 1994; Kuner et al., 1999; Bettler et al., 2004; Vanhoose et al., 2004; Connelly et al., 2013) and is in the same cell as the opioid receptors would have modulated GAT-1 activity. Although GABA_B_ receptors may modulate GAT-1 activity under other conditions or in different cells it does not occur in the PAG neurons important for opioid withdrawal.

The opioid sensitive GAT-1 activity in the PAG during withdrawal initiates the opioid withdrawal syndrome (Bagley et al., 2011). The GABA_B_ agonist baclofen reduces some of the signs of opioid withdrawal (Ahmadi-Abhari et al., 2001; Bexis et al., 2001; Tyacke et al., 2010) but does not alter the GAT-1 activity. One possible explanation for this is that an important outcome of the elevated GAT-1 activity during withdrawal is depolarization of GABA neurons and a resultant increase in synaptic GABA release. The increased GABA release inhibits PAG output neurons, changes neurotransmitter release in their target brain regions and ultimately expression of the withdrawal signs. Whilst GABA_B_ receptor activation can't reduce the GAT-1 activity that drives GABA release it could act to inhibit the excitability of GABAergic neurons through other mechanisms. Reduced GABA release could result from inhibition of GABA neuron excitability, through activation of GIRK, and inhibition of GABA release through non-GAT1 effectors. Through this alternative mechanism of inhibiting GABA release it would diminish inhibition of output neurons and thus withdrawal.

### Conflict of interest statement

The author declares that the research was conducted in the absence of any commercial or financial relationships that could be construed as a potential conflict of interest.

## References

[B1] Ahmadi-Abhari S. Akhondzadeh S. Assadi S. Shabestari O. Farzanehgan Z. Kamlipour A. (2001). Baclofen versus clonidine in the treatment of opiates withdrawal, side-effects aspect: a double-blind randomized controlled trial. J. Clin. Pharm. Ther. 26, 67–71 10.1046/j.1365-2710.2001.00325.x 11286609

[B2] Avidor-Reiss T. Nevo I. Saya D. Bayewitch M. Vogel Z. (1997). Opiate-induced adenylyl cyclase superactivation is isozyme-specific. J. Biol. Chem. 272, 5040–5047 10.1074/jbc.272.8.5040 9030567

[B3] Bagley E. Chieng B. Christie M. Connor M. (2005a) Opioid tolerance in periaqueductal gray neurons isolated from mice chronically treated with morphine. Br. J. Pharmacol. 146, 68–76 10.1038/sj.bjp.0706315 15980868 PMC1576256

[B4] Bagley E. Gerke M. Vaughan C. Hack S. Christie M. (2005b). GABA transporter currents activated by protein kinase A excite midbrain neurons during opioid withdrawal. Neuron 45, 433–445 10.1016/j.neuron.2004.12.049 15694329

[B5] Bagley E. Hacker J. Chefer V. Mallet C. McNally G. Chieng B. (2011). Drug-induced GABA transporter currents enhance GABA release to induce opioid withdrawal behaviours. Nat. Neurosci. 14, 1548–1554 10.1038/nn.2940 22037500

[B6] Beckman M. L. Bernstein E. M. Quick M. W. (1998). Protein kinase C regulates the interaction between a GABA transporter and syntaxin 1A. J. Neurosci. 18, 6103–6112 9698305 10.1523/JNEUROSCI.18-16-06103.1998 PMC6793212

[B7] Beckman M. L. Quick M. W. (1998). Neurotransmitter transporters: Regulators of function and functional regulation. J. Membr. Biol. 164, 1–10 10.1007/s002329900388 9636239

[B8] Bettler B. Kaupmann K. Mosbacher J. Gassmann M. (2004). Molecular structure and physiological functions of GABAB receptors. Physiol. Rev. 84, 835–867 10.1152/physrev.00036.2003 15269338

[B9] Bexis S. Ong J. White J. (2001). Attenuation of morphine withdrawal signs by the GABA(B) receptor agonist baclofen. Life Sci. 70, 395–401 10.1016/S0024-3205(01)01485-0 11798009

[B10] Chieng B. Bekkers J. (1999). GABA(B), opioid and alpha2 receptor inhibition of calcium channels in acutely-dissociated locus coeruleus neurones. Br. J. Pharmacol. 127, 1533–1538 10.1038/sj.bjp.0702693 10455306 PMC1566140

[B11] Chieng B. Christie M. (1996). Local opioid withdrawal in rat single periaqueductal gray neurons *in vitro*. J. Neurosci. 16,7128–7136 8929422 10.1523/JNEUROSCI.16-22-07128.1996 PMC6578948

[B11a] Chieng B. Christie M. J. (1995). Hyperpolarization by GABAB receptor agonists in mid-brain periaqueductal gray neurones *in vitro*. Br. J. Pharmacol. 116, 1583–1588 10.1111/j.1476-5381.1995.tb16376.x 8564222 PMC1908916

[B12] Collier H. O. Roy A. C. (1974). Morphine-like drugs inhibit the stimulation by E prostaglandins of cyclic AMP formation by rat brain homogenate. Nature 248, 24–27 10.1038/248024a0 4361995

[B11b] Collis L. (2008). NSW Drug and Alcohol Withdrawal Clinical Practice Guidelines. Sydney, NSW: Better Health Centre Publications Warehouse

[B13] Connelly W. Fyson S. Errington A. McCafferty C. Cope D. Di Giovanni G. (2013). GABAB receptors regulate extrasynaptic GABAA receptors. J. Neurosci. 33,3780–3785 10.1523/JNEUROSCI.4989-12.2013 23447590 PMC3601669

[B14] Cristóvão-Ferreira S. Vaz S. Ribeiro J. Sebastião A. (2009). Adenosine A2A receptors enhance GABA transport into nerve terminals by restraining PKC inhibition of GAT-1. J. Neurochem. 109, 336–347 10.1111/j.1471-4159.2009.05963.x 19200339

[B15] Fedynyshyn J. P. Lee N. M. (1989). μ type opioid receptors in rat periaqueductal gray-enriched P2 membrane are coupled to G-protein-mediated inhibition of adenylyl cyclase. FEBS Lett. 253, 23–27 10.1016/0014-5793(89)80921-4 2547657

[B16] Gerber U. Gähwiler B. (1994). GABAB and adenosine receptors mediate enhancement of the K+ current, IAHP, by reducing adenylyl cyclase activity in rat CA3 hippocampal neurons. J. Neurophysiol. 72, 2360–2367 7884464 10.1152/jn.1994.72.5.2360

[B17] Guastella J. Nelson N. Nelson H. Czyzyk L. Keynan S. Miedel M. C. (1990). Cloning and expression of a rat brain GABA transporter. Nature. 249, 1303–1306 1975955 10.1126/science.1975955

[B18] Himmelsbach C. K. (1943). With reference to physical dependence. Fed. Proc. 2, 201–203

[B19] Kuner R. Köhr G. Grünewald S. Eisenhardt G. Bach A. Kornau H. (1999). Role of heteromer formation in GABAB receptor function. Science 283, 74–77 10.1126/science.283.5398.74 9872744

[B20] Laviv T. Vertkin I. Berdichevsky Y. Fogel H. Riven I. Bettler B. (2011). Compartmentalization of the GABAB receptor signaling complex is required for presynaptic inhibition at hippocampal synapses. J. Neurosci. 31, 12523–12532 10.1523/JNEUROSCI.1527-11.2011 21880914 PMC6703276

[B21] Maldonado R. Valverde O. Garbay C. Roques B. P. (1995). Protein kinases in the locus coeruleus and periaqueductal gray matter are involved in the expression of opiate withdrawal. Naunyn Schmiedebergs Arch. Pharmacol. 352, 565–575 10.1007/BF00169392 8751087

[B22] Margeta-Mitrovic M. Mitrovic I. Riley R. C. Jan L. Y. Basbaum A. I. (1999). Immunohistochemical localization of GABA_B_ receptors in the rat central nervous system J. Comp. Neurol. 405, 299–321 10076927 10.1002/(sici)1096-9861(19990315)405:3<299::aid-cne2>3.0.co;2-6

[B23] Mattick R. P. Hall W. (1996). Are detoxification programs effective? Lancet 347, 97–100 10.1016/S0140-6736(96)90215-9 8538351

[B25] Punch L. J. Self D. W. Nestler E. J. Taylor J. R. (1997). Opposite modulation of opiate withdrawal behaviors on microinfusion of a protein kinase A inhibitor versus activator into the locus coeruleus or periaqueductal gray. J. Neurosci. 17, 8520–8527 9334424 10.1523/JNEUROSCI.17-21-08520.1997 PMC6573752

[B26] Quick M. W. (2002). Substrates regulate γ-aminobutyric acid transporters in a syntaxin 1A-dependent manner. Proc. Natl. Acad. Sci. U.S.A. 99, 5686–5691 10.1073/pnas.082712899 11960023 PMC122832

[B27] Sharma S. K. Klee W. A. Nirenberg M. (1975). Dual regulation of adenylate cyclase accounts for narcotic dependence and tolerance. Proc. Natl. Acad. Sci. U.S.A. 72, 3092–3096 10.1073/pnas.72.8.3092 1059094 PMC432926

[B28] Terwilliger R. Z. Beitner-Johnson D. Sevarino K. A. Crain S. M. Nestler E. J. (1991). A general role for adaptations in G-proteins and the cyclic AMP system in mediating the chronic actions of morphine and cocaine on neuronal function. Brain Res. 548, 100–110 10.1016/0006-8993(91)91111-D 1651140

[B29] Tyacke R. J. Lingford-Hughes A. Reed L. J. Nutt D. J. (2010). GABA_B_ receptors in addiction and its treatment. Adv. Pharmacol. 58, 373–396 10.1016/S1054-3589(10)58014-1 20655489

[B30] Vanhoose A. Ritchie M. Winder D. (2004). Regulation of cAMP levels in area CA1 of hippocampus by Gi/o-coupled receptors is stimulus dependent in mice. Neurosci. Lett. 370, 80–83 10.1016/j.neulet.2004.07.093 15489022

[B31] Vaughan C. Bagley E. Drew G. Schuller A. Pintar J. Hack S. (2003). Cellular actions of opioids on periaqueductal grey neurons from C57B16/J mice and mutant mice lacking MOR-1. Br. J. Pharmacol. 139, 362–367 10.1038/sj.bjp.0705261 12770941 PMC1573857

[B32] Vaughan C. Ingram S. Connor M. Christie M. (1997). How opioids inhibit GABA-mediated neurotransmission. Nature 390, 611–614 10.1038/37610 9403690

[B33] Vigot R. Barbieri S. Bräuner-Osborne H. Turecek R. Shigemoto R. Zhang Y. (2006). Differential compartmentalization and distinct functions of GABAB receptor variants. Neuron 50, 589–601 10.1016/j.neuron.2006.04.014 16701209 PMC3531664

[B34] Whitworth T. L. Quick M. W. (2001). Substrate induced regulation of γ-aminobutyric acid transporter trafficking requires tyrosine phosphorylation. J. Biol. Chem. 276, 42932–42937 10.1074/jbc.M107638200 11555659

[B35] Williams J. T. Christie M. J. Manzoni O. (2001). Cellular and synaptic adaptations mediating opioid dependence. Physiol. Rev. 81, 299–343 11152760 10.1152/physrev.2001.81.1.299

